# Evaluating the Role of Seed Treatments in Canola/Oilseed Rape Production: Integrated Pest Management, Pollinator Health, and Biodiversity

**DOI:** 10.3390/plants5030032

**Published:** 2016-08-03

**Authors:** Gregory Sekulic, Curtis B. Rempel

**Affiliations:** 1 Canola Council of Canada, 400-167 Lombard Ave, Winnipeg, MB R3B 0T6, Canada; sekulicg@canolacouncil.org; 2 Faculty of Agricultural and Food Sciences, University of Manitoba, Ellis Building, Winnipeg, MB R3T 2N2, Canada

**Keywords:** canola, oilseed rape, honeybee, pollinator, integrated pest management, IPM, ecosystem, seed treatment, neonicotinoid

## Abstract

The use patterns and role of insecticide seed treatments, with focus on neonicotinoid insecticides, were examined for canola/oilseed rape production in Canada and the EU. Since nearly all planted canola acres in Western Canada and, historically, a majority of planted oilseed acres in the EU, use seed treatments, it is worth examining whether broad use of insecticidal seed treatments (IST) is compatible with principles of integrated pest management (IPM). The neonicotinoid insecticide (NNI) seed treatment (NNI ST) use pattern has risen due to effective control of several early season insect pests, the most destructive being flea beetles (*Phyllotreta sp.*). Negative environmental impact and poor efficacy of foliar applied insecticides on flea beetles led growers to look for better alternatives. Due to their biology, predictive models have been difficult to develop for flea beetles, and, therefore, targeted application of seed treatments, as part of an IPM program, has contributed to grower profitability and overall pollinator success for canola production in Western Canada. Early evidence suggests that the recent restriction on NNI may negatively impact grower profitability and does not appear to be having positive impact on pollinator health. Further investigation on impact of NNI on individual bee vs. hive health need to be conducted. Predictive models for flea beetle emergence/feeding activity in canola/oilseed rape need to be developed, as broad acre deployment of NNI seed treatments may not be sustainable due to concerns about resistance/tolerance in flea beetles and other pest species.

## 1. Introduction

This workshop was held as part of the 14th International Rapeseed Congress (IRC) held at TCU Place (Saskatoons’s Art and Convention Centre) in Saskatoon, Saskatchewan, Canada, 5–9 July 2015. The conference was co-organized by Ag-West Bio Inc. (www.agwest.sk.ca) and the Canola Council of Canada (www.canolacouncil.org). The Congress program comprised five Congress keynote presentations, 30 thematic keynote presentations, 31 concurrent oral sessions (four to five speakers in each) with a total of 143 speakers selected from the approximately 500 abstracts received. Approximately 300 posters were displayed and nine workshops on special topics were organized (see http://gcirc.org/event/single-view/article/14th-international-rapeseed-congress-july-2015.html). The workshops provided opportunity for researchers to present their findings, discuss and debate their work, and exchange viewpoints and information.

This workshop examined the use patterns of insecticide seed treatments, with focus on neonicotinoid insecticides (hereafter referred to as NNI or NNI ST), in canola/oilseed rape production in Western Canada and the EU. Since nearly all of the planted canola acres in Western Canada use seed treatments, it is worth examining whether treatment of 99.5% of acres with an insecticidal seed treatment (IST) is compatible with the principles of integrated pest management (IPM). Canola growers are focused on optimizing crop yield and quality at maximum profitability, both for their farm and the value chain, and this can include health of natural and managed pollinators and other arthropods present in and around the crop. Furthermore, the workshop provided discussion and perspective on two questions that are germane to successful (IPM) practices in the future. These are:

(1) Have seed treatments, as part of an IPM programme, led to more profitable canola crops, healthy pollinator populations and advanced arthropod biodiversity in canola/oilseed rape?
(2) Will seed treatments continue to be an important component of IPM in the future?

The workshop opened with a discussion on general principles of IPM and the importance of IPM for canola/OSR production globally. The consensus among workshop participants—consistent with the weight of evidence from the scientific literature—is that IPM in canola/OSR cropping systems has:

- Achieved effective management of pests by using all tools available in the safest manner possible.
- Utilized cultural, mechanical, biological and chemical control measures in a harmonized fashion to strive for balance of profitability, sustainability, and efficacy.
- Enhanced the economic viability of farms.

### Workshop Topics and Presenters

- Gregory Sekulic—Canola Council of Canada (CCC), Grande Prairie, AB Canada: “Evaluating the role of seed treatments in an Integrated Pest Management regime”.
- Dr. Michael Schade—Syngenta Crop Protection, Basel, Switzerland: “Impact of neonicotinoid suspension in EU28”.
- Caroline Nicholls, Agriculture and Horticulture Development Board (AHDB) Cereals and Oilseeds, Kenilworth, Warwickshire, UK: “Growing winter oilseed rape without neonicotinoid seed treatments—the UK perspective”.
- Dr. Udo Heimbach (U. Heimbach, M. Stähler, D. Schenke, A. Dietzsch, N. Kunz, I.P. Wurz, J. Pistorius), Julius Kühn-Istitut, Federal Research Centre for Cultivated Plants, Braunschweig, Germany: “Impacts of neonicotinoid use in oilseed rape and their mitigation.”
- Dr. Nicolas Cerrutti (N. Cerrutti, C. Rüger, M. Gayrard, J.-F. Odoux, P. Aupinel, A. Decourtye. M. Henry, V. Bretagnolle), Terres Inovia, ETOILE sur RHONE, France: “Impact of neonicotinoid insecticide thiamethoxam on the lifespan of the honeybee *Apis mellifera* L. in a large scale study conducted under natural conditions”.
- Sreten Terzić (Željko Milovac, Filip Franeta, Ljubiša Stanisavljević, Sreten Terzić, Ana Marjanović Jeromela, Velimir Radić), Institute of Field and Vegetable Crops, University of Belgrade, Novi Sad, Serbia: “Using Mason bees for rapeseed yield improvement”.

NOTE: Every researcher submitting an abstract following public request for papers by the IRC organizing committee presented at the workshop. This workshop report contains the research findings and observations that were presented by the speakers, the literature that was highlighted in the presentations and discussions, as well as the contents of discussions following the presentations and round-table forum.

## 2. Definition and Principles of Integrated Pest Management (IPM)

Authors Note: Due to time limitation for the workshop, and the presenters and participant’s backgrounds in Integrated Pest Management, there was unanimous consensus not to spend time on definitions or principles of IPM. To guide the reader, we will briefly highlight some of the principles of IPM that are germane to this workshop.

IPM is the careful consideration of all available pest control techniques and subsequent integration of appropriate measures that discourage the development of pest populations and keep pesticides and other interventions to levels that are economically justified and reduce or minimize risks to human health and the environment [1]. IPM programs for canola combine cultural controls, such as planting dates, increased seeding rates, as well as seed treatments to ensure adequate populations of vigorous plants [2]. There are two important considerations for growers in managing early season pests: Preserve the targeted plant population and protect the overall health of the surviving plants.

A description and tools for implementation of IPM in canola for western Canada is found at http://www.canolacouncil.org/crop-production/canola-grower’s-manual-contents/chapter-10-integrated-pest-management/chapter-10#canolaintegratedpestmanagement. A central tenet of IPM is the establishment of action thresholds to determine when a pest population warrants control; these thresholds do not exist for many of the pests that are controlled by seed treatments and even when they do, the availability of grower-viable testing is extremely challenging. Research efforts are needed to develop and test pest predictor models that will allow growers to reliably monitor their fields and surrounding habitats in order to make informed decisions about the need to use insecticide and/or fungicide treated seed. However, in absence of these models, it is an infringement of IPM principles to limit growers’ access to the only tool that can protect the crop. This forces growers to use more costly and less efficacious foliar insecticides that have higher toxicity to non-target species.

## 3. History and Evaluation of Seed Treatment Insecticide Use in Canola in Canada

The neonicotinoid insecticide seed (NNI) treatment use pattern has primarily evolved to prevent losses due to crucifer flea beetles and striped flea beetle (Phyllotreta cruciferae, Phyllotreta striolata) during emergence and establishment in the weeks following planting of the crop [3]. The crop is particularly vulnerable to yield loss at this stage. Flea beetles are the most economically damaging insect pests of canola on the Canadian prairies [3]. Yield losses due to flea beetles were in the range of 8%–10%/annum and resulted in $130–300 million/annum in lost farm income [4,5,6]. Severe economic damage typically occurs in the spring through defoliation of canola seedlings by adult flea beetles emerging from overwintering. Foliar feeding often leads to yield reductions of 10%–50% when flea beetles are present at medium to high infestation levels [7]. For the 2014 crop year 8,407,200 ha were planted to canola with production of 16,410,000 tonnes. The price of canola on 2 September 2014, Vancouver was $464/tonne. Thus, failure to control damage by flea beetles resulted in a potential loss of $760,960,000 to the Canadian rural economy [8].

The neonicotinoid class of insecticides—which includes imidacloprid, clothianidin, and thiamethoxam—was introduced in the mid-1990s. They have been well-received by farmers, regulators and ecotoxicologists due to their novel mode of action, efficacy against many serious insect pests and selectivity for insects over vertebrates. Due to their systemic activity, the neonicotinoids can be applied to seeds or soils at low rates and provide extended protection to crops during their vulnerable early growth stages. This reduces the number of foliar insecticide applications required, which are applied at much greater application rates and generally pose more hazards to non-target organisms. Neonicotinoid insecticides specifically target the insect nicotinyl receptor, which is structurally different from the mammalian receptor. Consequently, their mammalian toxicity is much lower than the products that they have replaced [9]. Prior to the development of NNI, lindane was used as a seed treatment, or in-furrow or foliar applied insecticides were used. These generally had high mammalian toxicity and significant non-target effects on pollinators, ground beetles, spiders, and other beneficial insects.

While occurrence of striped flea beetles and other flea beetle species found in prairie-wide surveys is increasing, the crucifer flea beetle still remains the predominant species in fields and is found in all areas of Western Canada where canola is produced and in high numbers, numbers which typically exceed an economic threshold [10].

The economic or damage threshold for the canola cotyledon or leaves is based on leaf area loss. The economic threshold for canola is 25% loss of leaf area of individual plants, at which control measures should be initiated [11]. While young seedlings often cannot compensate for extensive damage, once the crop is past the four-leaf stage, historic data indicate that, for spring-sown canola in Western Canada, significant losses do not occur as a result of flea beetle feeding [11]. Yield loss due to flea beetle injury is highest during the first one to three weeks after crop emergence and yield loss can be minimized if adult beetles are controlled during those weeks [7]. Most importantly, on warm days, flea beetle feeding and concomitant leaf damage can advance from 25% to 50% within hours [12]. Under high flea beetle pressure (linked to suitable environmental conditions) feeding can occur so rapidly that delaying treatment by 1–2 days can result in loss of the entire field [6]. Nearly every year on the Canadian prairies, farmers have to replant canola fields due to catastrophic loss from flea beetle feeding. The number of fields and geographic distribution is highly variable from year to year. Consensus among growers, extension agronomists, entomologists and scientists is that losses would be significantly greater and significantly higher volume and frequency of foliar insecticides would be applied if NNI ST were not used as a broad acre component of IPM. The widespread occurrence and rapidity that flea beetle feeding can go from little damage to catastrophic loss make the continued use of NNI ST necessary for a healthy, profitable canola crop. NNI ST provides protection against flea beetles during the critical period (0–21 days) of plant emergence and establishment.

NNI ST has been shown to protect or increase yields of canola when exposed to flea beetles. Research data has shown that research plots planted with insecticide seed treatment have 6%–43% more plants (av. 24%) with concomitant 0%–46% yield increase (av. 16%) over non-treated plots [13]. Nonetheless, and despite widespread use, insecticide seed treatments do not provide sufficient protection against significant economic loss when flea beetle populations are high and soil temperatures and ambient air temperature are cool. Under these conditions, the active ingredient is not taken up and translocated by the plant at a sufficient rate to inhibit flea beetle activity and prevent leaf damage. This situation occurs relatively frequently in parts of Western Canada with flea beetle outbreaks documented in both 2012 and 2014 necessitating the use of 2–3 separate foliar insecticide applications as rescue treatments on approximately 15% of canola acres. This can negatively impact profitability and arthropod biodiversity. Frequent and repeated use of foliar insecticides may negatively affect non-target insects such as pollinators and parasitoids [14]. When a seed treatment is used, the insecticide is applied prior to planting and consequently they are a viable option for reducing exposure to insecticides in non-target beneficial insect populations [15].

Applications of foliar insecticides are not necessarily harmful to bees early in the season. They do most damage to carabid and rove beetles that prey on root maggots. The Canola Council of Canada agronomists, provincial field entomologists, and disease specialists are observing significant late season root maggot damage and yield losses in canola fields that have used foliar insecticides for control of flea beetles. Research is currently underway (administered by CCC) to understand the impact on preserving biodiversity in canola crops and IPM practices that reduce risk to beneficial insects including pollinators.

Ideally, growers would like predictive models that could predict flea beetle emergence and activity prior to canola planting. Development of a flea beetle prediction model would help to guide growers to purchase/forego seed treated with IST or apply foliar insecticides only when appropriate. Despite significant historical investment in research, a functional predictive model has not been forthcoming [3,10,16,17,18,19,20]. Difficulty in monitoring overwintering populations, a wide window for emergence, rapid movement/high mobility, and aggressive feeding habits are reasons why predictive models have been difficult to develop.

To prevent significant annual yield losses from flea beetle outbreaks and to mitigate the high economic and environmental costs of multiple foliar insecticide applications, agronomists and entomologists in Canada have therefore agreed that an appropriate IPM program for canola includes the use of an IST on all planted canola acres [13].

## 4. Non-Target Impact of Seed Treatment Insecticide Use on Canola

Seed treatment insecticides are systemic, which allows them to move from the treated seed into the young growing roots and leaves of the seedling and confer protection to the young plant for up to 40 days post emergence. Systemic insecticides move via the xylem to the leaves of the plant; transport back to other parts of the plant, including flowers, can only occur via the phloem and studies indicate that very little is transported this way [21,22]. As a result, residue levels in nectar and pollen are expected to be low. This is corroborated by existing data that confirm exposure via pollen and nectar of treated crops is negligible [23,24,25,26]*.* Nonetheless, numerous groups have questioned the potential for non-target insects, especially pollinators, to be exposed to seed treatment insecticides via the pollen and nectar of plants grown from treated seeds. This has led to numerous questions regarding the validity of using seed treatment insecticides as a preventative treatment and raised the profile of IPM among a wide range of stakeholder groups.

While seed treatment insecticides are used on a broad range of field crops across Canada—including canola, alfalfa, mustard, potato seed, cereals, lentils, peas, chickpeas, and soy—canola is perhaps the best model system to evaluate the potential impacts of seed treatment insecticides on beneficial and non-target species. This is for a number of reasons, some of which will be discussed briefly below.

**Figure 1:**
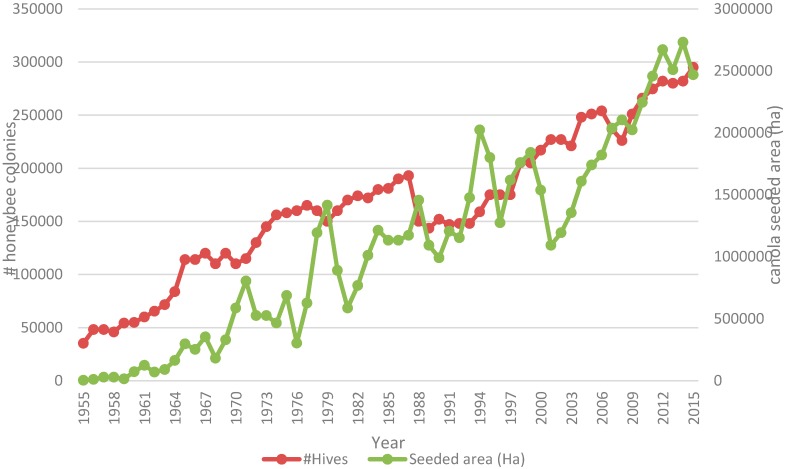
Historical canola planted acres and managed honeybee hives in Canada (1995–2015).

Canola is extremely attractive to bees [27] and represents a primary forage crop for honeybees in Western Canada where it is grown on 18–21 million acres annually. Field surveys examining neonicotinoid insecticide concentrations in canola pollen in Alberta (pollen is the protein source for bees) indicate that residue was detected in 32% of samples at an average concentration of 1.06 µg/kg (1 part per billion). These data are consistent with those reported from a 2012 production scale study of five NNI treated and five untreated fields where researchers found NNI pollen residue from 0.5 to 1.1 ppb and observed found no evidence of increased mortality or altered behavior of bees during planting or associated with foraging on seed-treated canola [9]. At a macro-scale, number of managed honey bee colonies has continued to increase in Western Canada concurrent with an increase in canola acreage from 6.25 to 20.09 million in the period from 1990 to 2015. (STATSCAN CANSIM Table 001-0017—Figure 1).

Pollen and nectar exposure studies were conducted in 5 field or semi-field locations planted to winter oilseed rape treated with clothianidin (10 g·a.i/ha) and beta-cyfluthrin (2 g·a.i/ha). Residues in nectar and pollen were measured and ranged from 0 to 2.9 ppb (mean 1.6 ± 0.43 nectar; 1.1 ± 0.37 pollen) [12]. No effects were found from exposure to nectar and pollen on honeybees, bumble bees, or *Osmia* sp., although it is worth noting sample sizes in this study were not always sufficient to permit residue detection. Residues from NNI in winter-sown oilseed rape pollen and nectar did not appear to affect bees but summer oilseed rape needs to be evaluated as well since seeding rates are higher for spring-sown oilseed rape and this may have an effect on exposure levels [28].

A French study indicated that ingestion of sublethal doses of thiamethoxam by honeybees could impact homing ability in semi-field tests [29]. Homing flight is a criterion used to assess sublethal effects of compounds on bees since it is a complex behavior that requires numerous biological processes including memory, motor function, orientation and flight [29]. A follow-up study conducted in 2013 and 2014 looked at free-ranging honeybees exposed to thiamethoxam (trade name Cruiser) as they foraged in an oilseed rape-dominant environment in western France [30]. In that study, researchers tagged 10,000 honeybees and studied the impact of thiamethoxam exposure on lifespan and homing behaviour on individual bees and the downstream impacts on colony viability. The researchers concluded that exposure to thiamethoxam under field-realistic conditions did result in reduced lifespan and increased homing failure in honeybees; however, these effects had no negative impact at the colony level.

Researchers at the Julius Kühn-Institut (JKI) in Germany have been investigating persistence and exposure of bees to NNI via guttation water, pollen, and nectar. Guttation is the excretion of droplets of xylem liquid at the tips or edges of leaves and it occurs more frequently in monocotyledon plants than in dicotyledons [28]. Guttation occurs when plants are sufficiently hydrated and transpiration is limited or inhibited (e.g., it can be triggered by relative humidity greater than 75%). While the risk of exposure of bees to neonicotinoids via guttation water was always assumed to be low, residues are detected in guttation at sufficiently high concentrations to warrant further research. Researchers at JKI conducted three field trials and 1 semi-field trial in winter oilseed rape looking at potential effects from exposures to guttation water and reported no observed impacts on colony development (including bee brood) or acute bee mortality [28]. The researchers observed that single bees collecting guttation drops in early development stages of winter oilseed rape could be killed or damaged if residue was present (LD_50_ oral toxicity = 3.7 ng/bee) but that risk is reduced significantly if sufficient water is available to the colony and bee hives are not placed directly in fields with emerging oilseed rape.

Biodiversity in canola fields is currently very high. Over 200 species of ground beetles, greater than 800 species of spiders, numerous *Staphylinids* (rove beetles), and at least 400 species of carabid beetles are commonly found in Canadian canola agroecosystems [31]. Canola fields also play host to a diverse range of beneficial insects including more than 1400 identified species of parasitoid wasps (comprising 1160 *Ichneumonidae* and 250 *Braconidae*) [31]; numerous species of native pollinators; and native predators such as lacewings (*Chrysoperla* spp.), which prey on *Lygus* nymphs (*Lygus* spp. are a frequent pest of canola). There is tremendous ecological and economic value in promoting and preserving these species in canola canopies; broad scale foliar application of foliar insecticides would dramatically reduce this beneficial insect biodiversity.

In summary, the Western Canadian canola experience indicates that ISTs, as part of an IPM program, have not led to a reduction in populations of managed pollinators and beneficial insects. Honeybee numbers in Western Canada have increased; arthropod biodiversity in IST canola is strong; and canola plays host to numerous beneficial species including predators, parasitoids, and pollinators that all have utility for canola production. The key for sustainable agricultural production will be to expand our monitoring of both pest and beneficial insect species, develop predictive models for their occurrence and density and re-examine biological control of key canola pests within context of better understanding of the role of beneficial insects such as predators and parasitoids.

Furthermore, the use of insecticide seed treatments has helped to increase crop yield and profitability by facilitating earlier planting and producing a vigorous crop that is better able to handle environmental stresses. This has led to improved canopy cover and more flowers per plant [13]. NNI ST have been reported to enhance plant vigor and stress tolerance (both biotic and abiotic) and better tolerate pest pressure, independent of their insecticidal role [32,33,34,35,36], in part through induction of salicylate-associated plant defense responses [37]. The opportunity to forego foliar insecticide applications due to application of NNI ST has also resulted in lower costs. NNI ST have contributed directly to increased canola yield and profitability and have reduced production risk and this is a key reason for expanded canola acreage in Western Canada over the past decade [28].

## 5. Concerns about Seed Treatment Use in Other Crops and Dissimilarities for Canola/Oilseed Rape Production

In Germany (like Eastern Canada), bee mortality has been observed following exposure to dust released from planters during the planting of neonicotinoid-treated corn (maize) [28,38]. Corn has a rough, waxy coating which tends to repel NNI seed treatment more than the oily seed coat of oilseed rape that results in a significant amount of material that can readily move from the seed when handled.

**Figure 2:**
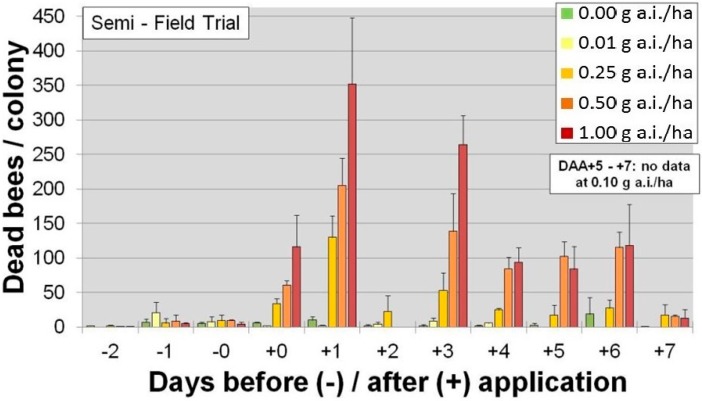
Honeybee mortality in a semi-field trial following manual application of dust fractions (<160 µm) length of time from application of different rates of clothianidin (g·a.i./ha) [28].

Corn seeds are also planted using precision, pneumatic planters that have air outlets and vent air externally into the surrounding environment. The precision planter ensures that there is optimal spacing between plants. Since corn seed is irregular in shape, they have a tendency to become stuck in the planting machinery so seed companies typically add a lubricant to the treatment or growers use a seedbox lubricant to prevent blockage. Until 2014, the lubricants of choice were either graphite or talc, both of which adsorbed traces of insecticide. When corn is sown during dry, windy conditions, the dust containing trace amounts of insecticide could be vented from the planter and, if bees are foraging in the vicinity, could result in inadvertent exposures. For example, Figure 2 shows the number of dead honeybees in traps in a semi-field trial following manual application of dust fractions (<160 µm) following differing rates of clothianidin (g·a.i./ha) [28].

When considering the potential risk to non-target organisms from fugitive dust emitted during the planting of NNI-treated seeds, it is necessary to remember that risk is a function of hazard and exposure. As such, it is important to consider the following variables:

(1) Emission of active ingredient. This is a function of seeding rate per ha x amount of dust abrasion x amount of active ingredient present in dust PLUS particle size, planter drill type, soil humidity, wind speed, and direction.
(2) Exposure. This is influenced by emission of active ingredient (see above); distance of the non-target organism from the point of emission (for example, see Figure 2); wind speed; wind direction; stickiness of surfaces that the dust may land on (e.g., plants); plant leaf architecture; and the physical and chemical properties of the active ingredient.
(3) Organism. The sensitivity and biology of the organism (e.g., presence, place, type and intensity of activity) influence whether the non-target species is likely to be exposed and if so, the nature of their physiological response to that exposure [28,38].

**Figure 3:**
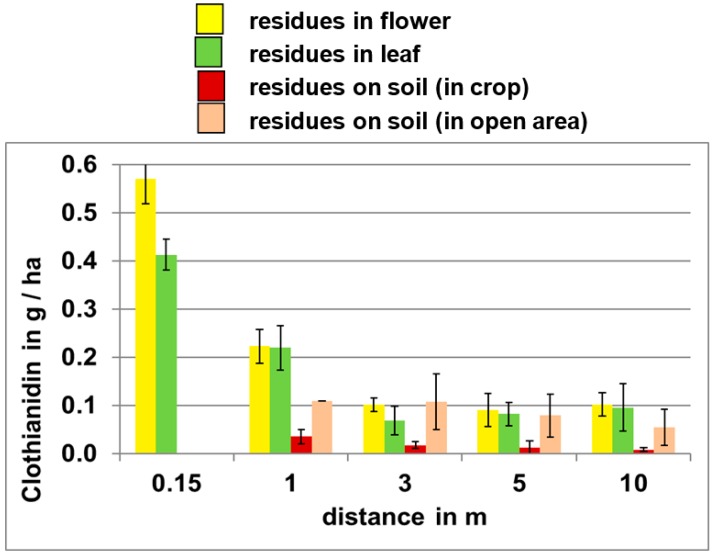
Clothianidin residue (g/ha) in oilseed rape crop or soil at various distances from the crop edge following sowing of corn (2010) [28].

The impact of distance from point of emission on potential exposure levels is illustrated in Figure 3, which shows clothianidin residues released during the planting of treated corn seed on oilseed rape plants and soil at various distances from the field edge. These data indicate that clothianidin residues on all media decrease with increasing distance from the site of emission.

Studies conducted with dust emitted from corn planters indicate that honeybees can be susceptible to fugitive dust containing NNI residues [39]. The levels of dust abraded from treated corn seeds are monitored using a Heubach Dustmeter; lower emissions, and therefore lower risks of exposure, are associated with Heubach values (expressed in g·a.i./ha). Exposure risks to non-target organisms in North America have been successfully reduced by limiting Heubach abrasion values; by using a wax-based fluency agent in place of talc or graphite; and by modifying planter design to minimize the amount of dust that is exhausted from the planter in the first place. It’s important to note that the risks associated with the planting of neonicotinoid-treated seeds have been limited to corn and soybeans; there are no reports of any issues with the planting of canola or oilseed rape. Furthermore, researchers studying the potential for dust released during planting of corn and soybean to affect bees foraging on winter-seeded oilseed rape have concluded that there are no unacceptable effects on honeybees if the seed treatment in corn is re-formulated to reduce dust.

The planting equipment used for sowing canola in Western Canada is different from equipment used to sow corn. Differences in planting equipment and the fact that the canola seed coat is “oily” and uniform (does not have the ridges and dents of corn or soy seed) means that there is no “caking” and “dust” dispersion of the IST from the canola planting operation. This results in significant risk reduction for bee/pollinator exposure to the NNI ST.

## 6. Insecticide Seed Treatment Use in the EU

In the European Union, NNI seed treatments (clothianidin, imidacloprid, and thiamethoxam (2–10 g·a.i./kg seed)) have been used to control major oilseed rape pests including cabbage flea beetle (*Phyllotreta* spp.), cabbage stem flea beetle (*Psylliodes chrysocephala*), peach-potato aphid (and other aphids as virus vectors—*Myzus persicae*), cabbage root fly (*Delia* brassicae) and turnip sawfly [16,18]. Similar to the advantages described above for Western Canada, since their introduction, NNI seed treatments in the EU have offered growers effective and extended pest control, coupled with low mammalian toxicity and low effects in the soil environment [28,40,41]. To date there are no reports of resistance to neonicotinoid insecticides for any of the target pests in oilseed rape (researchers in Canada have been monitoring for the past decade and tolerance to NNI is evident for striped flea beetle, *P. striolata*, in Western Canada [42]).

Crop establishment for a small seeded crop like canola or OSR is the foundation of crop management and yield security. Like Canadian canola growers, EU oilseed rape producers used insecticide seed treatments to protect the crop during its most vulnerable stage (cotyledon-4 leaf stage).

In recent years the use of insecticide seed treatment in the EU has been the subject of considerable debate. In April 2013, this culminated in the European Commission announcing a two-year moratorium on the use of imidacloprid, clothianidin, and thiamethoxam on all bee-attractive crops effective 1 December 2013 [32,43]. The effects of the ban on OSR cultivation were not expected to be evident until the 2015 growing season since much of the EU OSR crop is winter planted and therefore much of the 2014 harvested crop would have been grown from treated seed.

While opinions are mixed on the chain of events that led to this decision, it’s noteworthy that Dr. M. Flüh (Head, Chemicals Unit, EU Health and Consumers Directorate, EC) stated “the recent suspension of certain uses of NNI insecticides was not imposed because they are the main threat to bee health but because they were the only factor that could be quickly regulated by the EC” [38].

### 6.1. Impact of Neonicotinoid Restrictions in the EU

The restrictions on NNI in the EU28 are beginning to have an economic impact and therefore provide an opportunity to investigate potential impacts on pollinators and farm economics.

### 6.2. Impact on Growers

Growers of affected crops have reported farm income losses of up to approximately 5% and the results of an independent analysis found that NNI protected €2.1 billion ($3.1 billion CDN) of oilseed rape, corn, sugar beet, wheat, barley and sunflower crops across the EU and improved production efficiency by €0.7 billion ($1.03 billion CDN) [38]. The NNI restrictions introduced at the end of 2013 have resulted in reduced competitiveness of crop production; a reduction in crop diversity; an increase in Europe’s dependence on global imports for animal feed; and are putting 50,000 full-time jobs at risk [38].

Specific to oilseed rape production in some EU countries, the restrictions on NNI have resulted in a 6% decline in area planted to oilseed rape and farmers are reporting 90% of the planted crop is damaged by flea beetles, with 30% of this severely damaged [38,44]. In addition to substantially increased damage from flea beetle, farmers are facing higher input costs since they have to resort to foliar insecticide sprays to manage flea beetles. Across the EU, foliar applications have increased from two sprays up to 5.5 sprays/ha [38]. In the UK, foliar insecticide application has increased four-fold, reaching 100% treated ha in some regions [44]. This has resulted in costs that are now one quarter of the profit margin in some areas.

Oilseed rape producers in Germany report similar issues including no registered products for the control of cabbage root fly; limited registered options to control cabbage stem flea beetle; high risk of mortality for winter-seeded oilseed rape; reduced yield potential; and increased production risk [29]. Germany saw a five-fold increase of foliar pyrethroid insecticide applications in autumn 2014 compared to 2013 (prior to ban on NNI) and there are also reports of increased pyrethroid resistance in cabbage stem flea beetle. Germany saw a decrease in planted OSR acres in 2014 due to increased production risk (reduced yield security) and this trend is expected to continue [28]. Oilseed rape production has started to shift to Russia and the Ukraine, which now have a competitive advantage [32,34].

In addition to the yield loss, higher economic costs, and reduced profit margin from the adoption of foliar insecticide sprays, the restrictions on NNI use are also having a negative ecological impact. As farmers increase their reliance on pyrethroid foliar insecticides, agronomists and farm advisors anticipate insect resistance to pyrethroids will increase in the coming years [34]. This will mean that growers will run out of registered crop protection alternatives. As discussed earlier, foliar insecticide applications pose a higher risk to beneficial insects, and as a result it is reasonable to assume that secondary pests may also increase in significance. The use of targeted insecticide application via seed treatment is an important component in IPM because their use history clearly demonstrated a reduced likelihood of unintended negative consequences relative to the use of broadcast foliar insecticides.

**Figure 4:**
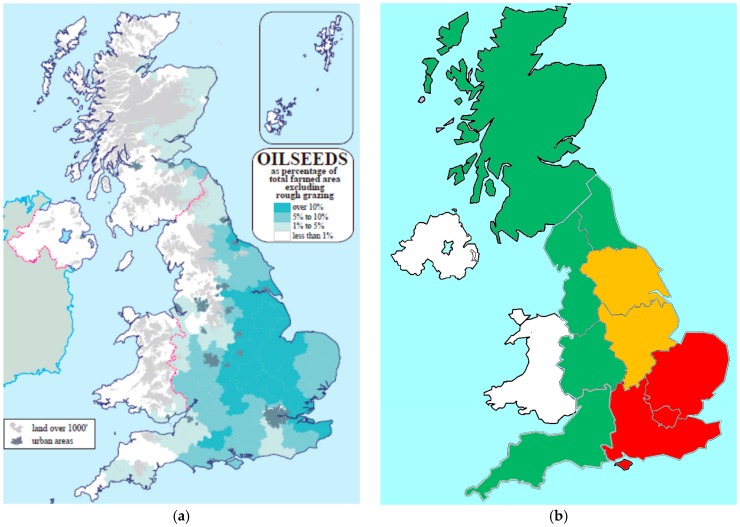
Incidence and severity of adult cabbage stem flea beetle in winter oilseed rape in the UK in 2015 [44] (**a**) Cropping Intensity (**b**) Insect severity. Red = Highest Loss and Risk.

In the UK, the area historically planted to winter oilseed rape treated with NNI was nearly identical to the area impacted by cabbage stem flea beetle, indicating growers were utilizing NNI as a key component in a pest management strategy. Figure 4 depicts the incidence and severity of adult cabbage stem flea beetle across the UK and indicates that they were highest in Eastern, South East, North East, and, Humber regions of the UK. Surveys across the UK confirmed the occurrence of pyrethroid resistant cabbage stem flea beetles in these regions.

In 2014, the greatest reduction in area not planted to winter oilseed rape due to NNI restrictions occurred in the eastern and southeastern regions and the highest losses due to cabbage stem flea beetle were reported in the eastern and southeastern regions. Areas in the UK with highest losses due to cabbage stem flea beetle infestation saw the largest reduction in oilseed rape planted area [34].

### 6.3. Impact on Pollinators

Impacts on pollinators (both native and managed as a result of the EU restrictions on neonicotinoids are harder to gauge since baseline data are limited and arguably lacking in scientific rigor. One important metric for measuring the health of honeybees is over-winter mortality. Historically, there has always been high spatial and temporal variability across European countries for over-winter bee mortality [45]. In winter 2012/2013 (i.e., the last year that NNI seed treatments were available and used), honeybee colony losses were particularly low; in contrast, in winter 2013/2014, reported losses were high [46]. In Sweden, in the first year of a field monitoring study (2014), they monitored 96 hives in total (48 were control) and found no difference in honey yield or winter survival between hives near crops grown from NNI-treated and control hives. Colony losses of up to 30% are historically common and can be explained by normal biological functions (Dr. Robin Moritz, Martin-Luther University, Halle-Wittenberg, IRC 2015). Researchers are not in agreement over the extent and pattern of colony losses across the EU.

Dr. Eike Genersch presented results of field studies during the Workshop on Bee Health at the Entomological Congress in Frankfurt, March 2015. The studies were designed to represent field-realistic exposure levels of bee colonies to crops grown from seeds treated with neonicotinoid seed treatments. These field-realistic studies are important to contextualize the data derived laboratory and greenhouse experiments that typically use artificially high exposures, which was the case with some of the studies described earlier in this paper. Dr. Genersch’s study monitored a cohort of more than 1200 bee colonies from 2005 to 2014, evaluating a number of different variables, including exposures to over 400 pesticides. Hives were selected for inclusion based on proximity to NNI-treated oilseed rape, sunflower, and cornfields and bee losses were compared to control hives that had no NNI exposure. The field study results indicated that there was no correlation between the pesticides studied and colony survival. These data from 10 years of official monitoring did find correlations between bee mortality, *Varroa destructor*, viral infections, and age of the Queen. Dr. Genersch stated that the most important conclusion from the study was that beekeepers who want to successfully bring their colonies through the winter season need to monitor and apply effective treatment for control of *Varroa* mite.

Researchers evaluated the role of Mason bees (*Osmia* spp.) for pollinating oilseed rape in Serbia (reported by Sreten Terzić). Mason bees are solitary species and each female forms its own nest. While the results of the research did not show significant oilseed rape yield increase due to presence of Mason bees, further research should be conducted as these bees are immune to *Varroa* mite [47].

## 7. Conclusions

While there are data to indicate that the use of neonicotinoid seed treatments in oilseed rape could have impacts on individual honeybees, the weight of evidence suggests that NNI ST alone are not sufficient to be the only negative impact on the health, productivity, or viability of the colony.

Risks to bees from the use of insecticides in general can be mitigated by minimizing the likelihood of exposure. Insecticide seed treatments are designed to be used in such a way that non-target organisms, such as bees and other beneficial insects, should never be exposed in the first place. They also use substantially less active ingredient per unit area than a foliar application, which reduces the likelihood of resistance development and minimizes the potential for damage to non-target and beneficial insects.

The nature of flea beetle biology is such that, to date, the development of a predictive model that would allow growers to predict precisely where an insecticide will be needed has been impossible to develop. As a result, growers must either use a seed treatment insecticide or plan to make multiple foliar applications of an insecticide to protect the developing plants from flea beetle attack. In Western Canada, growers have overwhelmingly chosen the former option. To date, this choice has not resulted in decline in honeybee colony numbers in the prairie provinces (populations continue to increase). Furthermore, canola growers, production agronomists, entomologists and bee keepers who utilize canola crops are increasingly realizing a strong and diverse ecosystem in and surrounding canola fields containing many species of beneficial insects. It is uncertain whether discontinued use of NNI ST and resulting reliance on older ST chemistry or increase foliar insecticide application would maintain or increase populations of these beneficial insects. In western Canadian canola production, use of NNI ST does not have a negative impact on pollinators and other beneficial insects.

NNI seed treatments are an essential part of our current effective IPM program in Canada. The EU experience indicates that the consequences of NNI restrictions for Canadian growers would be significant, including reduced profitability, reduced yields, increased planting risk, increased use of broadcast foliar insecticide applications, and increased insect resistance to pyrethroids.

Workshop participants agreed that the EU NNI restrictions need to be revisited with better scrutiny of the scientific data. The weight of evidence indicates that seed treatment insecticide use in OSR, when label requirements are adhered to, does not have a negative impact on honeybees. Furthermore, while the data are still limited, the evidence indicates that the European NNI restrictions have not had a positive impact on honeybee health; therefore, further restrictions would continue to negatively impact oilseed rape production with little or no benefit to honeybees.

While we can, and should, strive to develop models that allow us to use insecticides only where (and when) they are needed, in the meantime seed treatment insecticides in canola represent the most environmentally sustainable approach to protecting the crop and the environment, As such they play, and will continue to play for the foreseeable future, an important role in an IPM approach for canola and oilseed rape.

## Abbreviations

The following abbreviations are used in this manuscript:

DOAJ	Directory of open access journals
TLA	Three letter acronym
LD	linear dichroism
